# Irreversible electroporation of the liver: is there a safe limit to the ablation volume?

**DOI:** 10.1038/srep23781

**Published:** 2016-04-01

**Authors:** P. Sánchez-Velázquez, Q. Castellví, A. Villanueva, R. Quesada, C. Pañella, M. Cáceres, D. Dorcaratto, A. Andaluz, X. Moll, M. Trujillo, J. M. Burdío, E. Berjano, L. Grande, A. Ivorra, F. Burdío

**Affiliations:** 1 Department of Surgery, Hospital del Mar, Hospital del Mar Medical Research Institute (IMIM), Passeig Marítim 25-29, 08003, Barcelona, Spain; 2 Department of Information and Communication Technologies, Universitat Pompeu Fabra, Carrer Roc Boronat 138, 08018, Barcelona, Spain; 3 Translational Research Laboratory, Catalan Institute of Oncology (ICO), Bellvitge Biomedical Research Institute (IDIBELL), Av. de la Granvia de l’Hospitalet, 199-203, 08908 L’Hospitalet de Llobregat, Barcelona, Spain; 4 Department of Animal Medicine and Surgery, Faculty of Veterinary Medicine, Autonomous University of Barcelona (U.A.B), Plaza Cívica, s/n, 08193 Bellaterra, Barcelona, Spain; 5 Electronic Engineering Department, Universitat Politècnica de Valencia, Camino de Vera, 46022 Valencia, Spain; 6 Department of Electric Engineering and Communications, University of Zaragoza, Pedro Cerbuna, 12, 50018 Zaragoza, Spain

## Abstract

Irreversible electroporation is a fast-growing liver ablation technique. Although safety has been well documented in small ablations, our aim is to assess its safety and feasibility when a large portion of liver is ablated. Eighty-seven mice were subjected to high voltage pulses directly delivered across parallel plate electrodes comprising around 40% of mouse liver. One group consisted in 55 athymic-nude, in which a tumor from the KM12C cell line was grown and the other thirty-two C57-Bl6 non-tumoral mice. Both groups were subsequently divided into subsets according to the delivered field strength (1000 V/cm, 2000 V/cm) and whether or not they received anti-hyperkalemia therapy. Early mortality (less than 24 hours post-IRE) in the 2000 V/cm group was observed and revealed considerably higher mean potassium levels. In contrast, the animals subjected to a 2000 V/cm field treated with the anti-hyperkalemia therapy had higher survival rates (OR = 0.1, 95%CI = 0.02–0.32, p < 0.001). Early mortality also depended on the electric field magnitude of the IRE protocol, as mice given 1000 V/cm survived longer than those given 2000 V/cm (OR = 4.7, 95%CI = 1.8–11.8, p = 0.001). Our findings suggest that ionic disturbances, mainly due to potassium alterations, should be warned and envisioned when large volume ablations are performed by IRE.

*Electroporation (EP)* is a biophysical phenomenon becoming increasingly used as an ablation technique. Although the mechanism of EP is not yet fully understood, it is widely accepted that high electric field pulses delivered across tissues cause pores in the cell bilayer membrane thereby increasing its permeability. When these pulses are delivered with sufficient magnitude and duration, cell death is achieved and the process is defined as irreversible electroporation (IRE)^1^,^2^,^3^,^4^. IRE has shown complete responses and reduced tumor size after treatment of several solid tumors^5^,^6^,^7^,^8^,^9^,^10^.

As only 10–20% candidates for liver surgery are potentially resectable^11^, ablative techniques are perceived as promising alternatives and assessed in terms of their capability to ablate large volumes^12^. Until now, clinical IRE is typically performed in small tumors (diameter <3 cm). However, there are some pioneering studies indicating a growing interest in treating larger volumes. For instance, use of IRE in large tumor models was evaluated by Lee *et al*. who achieved a maximum ablated volume of 22.1 cm^3^ in a liver rabbit model (between 25–32% of total volume)^13^ with favourable outcomes^14^. Appelbaum *et al*. demonstrated that IRE application was clinically acceptable addressed to tumors >6 cm^15^.

Our group has been working on a novel IRE approach carried out in an immunosuppressed murine model of liver metastases in which the IRE ablation volume was unusually large^16^: we delivered pulses across two parallel plates that comprise two segments of the left liver which corresponds to nearly 40% of its volume^17^,^18^. Unlike thermal ablation techniques, IRE preserves the extracellular matrix and this may allow novel treatment paradigms. Our aim was to assess whether a large ablation could achieve the antitumor effect while preserving sufficient the liver parenchyma and function, in order to concomitantly ablate several nodules with a single applicator.

Safety of IRE has been extensively reported when moderate volumes are ablated^19^,^20^,^21^,^22^. However, our preliminary results showed high post-treatment mortality when larger volumes were treated.

We hypothesized that ablating such volume might had caused a massive release of intracellular contents into the bloodstream. We deemed that release of potassium was probably a major mortality cause as most deaths occurred a few minutes after delivery of the pulses. In the present study, to test this hypothesis, a subset of the animals was treated with intensive anti-hyperkalemia therapy. In addition, since we were concerned that immunosuppressed mice may be especially susceptible to IRE^23^, we conducted the same treatment in a subset of immunocompetent animals.

Thus, the aim of this study was to assess the safety and feasibility of IRE when large volume of liver is subjected to ablation.

## Materials and Methods

All aspects of this study were performed as part of an animal research protocol, in accordance with the guidelines approved by the Government of Catalonia’s Animal Care Committee (procedure FBP-13-1474, DAAM: 7016). These guidelines follow the *Directive 2010/63/EU* of the European Parliament and of the Council of 22 September 2010 on the protection of animals used for scientific purposes. All authors had control of the data and the information submitted for publication.

### Animal model

Two strains of six-week-old male mice (n: 87), immunodeficient athymic-nude (Harlan laboratories, Indianapolis, IN, USA) and immunocompetent C57-Bl6 (Charles River Laboratories, New York, NY, USA) were acquired and maintained under standard conditions with a laboratory diet and water ad libitum.

The groups were differentiated according to the IRE protocol applied, the mouse strain, and whether or not they received specific anti-hyperkalemia therapy (detailed in Table 1).

### Tumor implantation

The KM12C human colon cancer cell line was implanted in immunodeficent mice (n: 55). Tumors subcutaneously implanted and passaged in nude mice were extracted to prepare tumor fragments of 2 × 2 × 2 mm^3^ and then implanted. Animals were anesthetized with a mixture of isofluorane and inhaled oxygen and analgesia was provided with buprenorphine (0.05–0.1 mg/kg SC) and meloxicam (1–3 mg/kg SC). After anaesthesia, a subxiphoid laparotomy was performed and the left lobe of the liver exposed. The tumor was stitched to the hepatic capsule using a monofilament suture of 6/0 prolene. The abdominal muscle layer was closed with a running suture of vicryl 5/0, and the skin with a single silk suture.

Following initial implantation, 15 days were allowed for tumor growth. All the animals from the initial 55 implanted animals developed a tumor suitable for an IRE procedure and the treatment was applied after the growth period.

### Irreversible electroporation treatment

**Figure 1:**
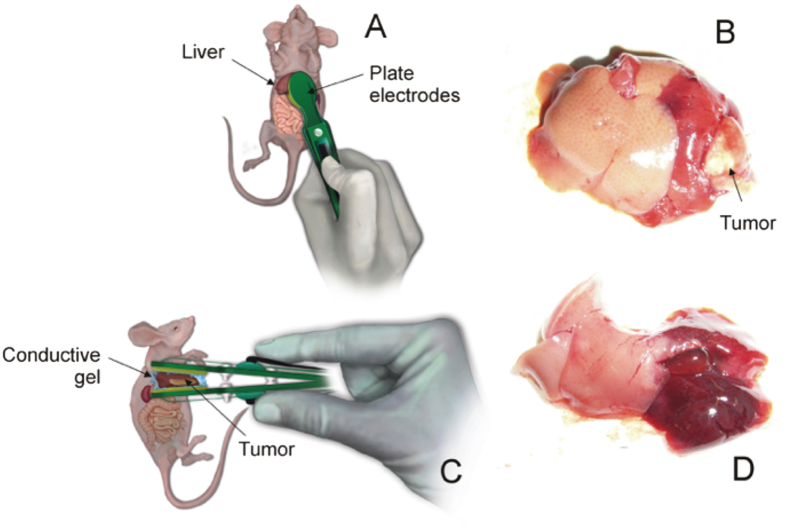
(**A,C**) Anterior and lateral view showing the plate electrodes placement on the targeted hepatic lobe in both strains of mice. (**B**) Photograph reveals the appearance of the electroporated left liver with the tumor in ID mice. (**D**) Hyperemic appearance of the left liver after the IRE in IC mice (without tumor).

After exposing the liver, plate electrodes were placed according to Fig. 1A,C. Not only the whole tumor was placed between the two parallel plate electrodes, but also both two segments of the left mouse liver, which represents around 40% of total size^24^. In contrast to needle electrodes, this approach ensures a quite uniform electric field across the treated volume thereby facilitating analysis. Furthermore, for further improving electric field uniformity a partially conductive gel (Aquasonic 100 Sterile, Parker Laboratories, Fairfield, NJ, USA) was applied between the two parallel plate electrodes^25^.

As a wide range of field magnitudes has been reported for complete ablation by IRE^26^,^27^, we evaluated two different magnitudes: 1000 V/cm and 2000 V/cm and a subset of animals were sham operated. The custom made generator was programmed to deliver ten sequences of ten pulses with a pulse duration of 100 μs and a repetition frequency of 1 Hz with a 10 s pause in between the ten pulses sequences.

During the first 72 h after surgery all the animals were given two additional doses of buprenorphine (0.05–1 mg/kg SC) and meloxicam (1–3 mg/kg SC), spaced out over 12 h increments.

### Pharmacological therapy against hyperkalemia

Prior to surgery, the set of animals corresponding to this treatment group (n: 42) were pre-hydrated with 200 μL of 0.9% NaCl (i.p.) solution every 24 h for two consecutive days. Calcium gluconate (GC) was injected in the preoperative care in two boluses, both intravenously (i.v.) and intraperitoneally (i.p.) at a dose of 100 mg/kg^28^. To prevent hyperkalemia, a furosemide i.v. bolus (4 mg/kg) was administered and during anaesthesia induction oxygen was mixed with inhaled salbutamol (100mcg). After the procedure, sodium bicarbonate (NaHCO_3_) i.p. was administered before the closure of the abdominal wall at a dose of 1 mEq/kg^28^.

Analytical tests at 10 min post-IRE were performed to quantify blood electrolytes in all the animals with an i-STAT® device (Abbott Point of Care Inc, Princeton, NJ, USA). Blood samples obtained by a single puncture in the saphena vein. Serum levels of sodium (Na^+^), potassium (K^+^), and HCO_3_^−^ were measured immediately after the extraction using the i-STAT®.

### Estimation of electroporated volume

The following parameters were evaluated in the liver samples harvested during the *in vivo* experiments: total liver weight, electroporated liver weight, total liver volume and electroporated liver volume. A precision scale was used to determine the weight. Volume was measured by placing the tissue samples in a laboratory graduated cylinder filled with water and quantifying the volume displaced.

### Statistical Analysis

All the statistics were processed by the SPSS statistical software package (SPSS, version 20, IBM, Armonk, NY, USA) and expressed as mean ± standard deviation.

The Kolmogorov–Smirnov test was used to determine the distribution of the variables. The Student’s t-test was used to make pairwise comparisons of normally distributed parameters and the Mann-Whitney U test was used for non-parametric data. The influence of hyperkalemia and IRE protocol on mortality was determined by univariable and multivariable logistic regression analyses. Results were expressed as Odds Ratio (OR) with 95% Confidence Interval (CI). Tests were considered statistically significant with a p-value of <0.05.

## Results

### Irreversible electroporation of a large hepatic volume produces severe hyperkalemia

Thirty-four from the total of electroporated animals (47.2%) died in the first 24 hours. Specifically, this early mortality occurred in 88% of the animals subjected to a 2000 V/cm field with no anti-hyperkalemia therapy (Table 1). There were no deaths during the application of the pulses, after which the animals were lethargic and tachypneic, presumably to compensate metabolic acidosis (Kussmaul breathing). Individuals showed lack of grooming and locomotion and eventually died (death times ranged from 5 minutes to 24 hours). At necropsy, hyperemia and vascular congestion were observed in the section of the liver that had received the pulses. However, no structural damage or intra-abdominal haemorrhages were noticed that could explain the early death of these animals. No other findings were encountered at the necropsies.

The serum potassium levels of all the animals were separately analyzed, considering 24-hour survival as a cut off value. Mean potassium levels were remarkably lower in the up to 24 h survival group (7.74 ± 1.7 mmol/L) than in the other group (9.49 ± 4.1 mmol/L), reaching statistical significance (p = 0.001). Logistic regression was applied to estimate the effect of hyperkalemia on survival. The crude odds ratio (OR) was 1.37 (95%CI = 1.07–1.73, p = 0.01), which indicated a significantly higher value of potassium in the animals that did not survive up to 24 h (Table 2). However, the group differences in sodium or HCO_3_ levels did not reach statistical significance.

The mean electroporated volume was 548.7 μL, which represented 37.9% of the mean liver volume (1448 μL). In terms of weight, the mean electroporated mass was 561.8 mg, or 38.6% of the mean liver mass (1456 mg).

### Early mortality depends on the electric field magnitude of the IRE protocol

As mentioned above, we observed that 88% of the animals that had not received anti-hyperkalemia treatment and given the highest intensity (2000 V/cm) died between 15 minutes to 24 hours after application. On the other hand, only 23.1% of those treated with 1000 V/cm protocol died in the first 24 h after the IRE-procedure and showed a similar symptomatology to those belonging to the groups treated at 2000 V/cm. The remaining mice of the 1000V/cm group (77.9%) achieved a longer overall median survival. The subset of sham-operated animals presented an uneventful postoperative course in the first 24 h, remaining active with normal food intake and no hunched posture. No further alterations were noticed.

Logistic regression was applied to estimate the effect of the different IRE magnitudes on early survival. The protocols were categorized considering two groups; those that had received the maximum intensity (2000 V/cm) and those that had not, again using the 24-hour cut-off value. The odds ratio (OR) of animals receiving 2000 V/cm protocol compared to the rest was 4.7 (95%CI = 1.8–11.8, p = 0.001), which indicates that receiving an IRE protocol of 2000 V/cm increased the risk of death by four times in the first 24 h post-IRE (Table 2).

### Management of electrolyte imbalances increases early survival after Irreversible Electroporation of a large volume of tissue

As shown in the previous section, the animals that received an IRE-protocol of 2000 V/cm had a poorer survival rate than the others. To ascertain the efficacy of the anti-hyperkalemia therapy, we analyzed separately the survival rate of the animals that received maximum voltage (2000 V/cm) plus anti-hyperkalemia therapy and those who did not receive the therapy.

Univariable logistic regression analyses revealed that the protocol 2000 V/cm animals that received the anti-hyperkalemia therapy had dramatically lower death rates in the first 24 h post-IRE than the other group (Table 2). (OR = 0.1; 95%CI = 0.02–0.32, p < 0.001).

### Multivariable model ROC curve

To further assess the contribution of hyperkalemia, the IRE protocol and anti-hyperkalemia therapy to the survival rate and to avoid potential confounders, all the significant variables from the univariable analyses were included in a multivariable model (Table 2). Three variables were statistically significant: (i) IRE protocol 2000 V/cm (OR = 17.8; 95%CI = 2.5–125.6, p < 0.004), (ii) postoperative hyperkalemia (OR = 1.4, 95%CI = 1.0–1.9, p < 0.028) and (iii) medical treatment against hyperkalemia (OR = 0.3, 95%CI = 0.1–0.8, p < 0.023). The area under the ROC curve was 0.9 (95%CI = 0.84–0.99, p < 0.001) for the logistic regression model, which reveals very good goodness-of-fit.

### Histopathological evaluation

**Figure 2:**
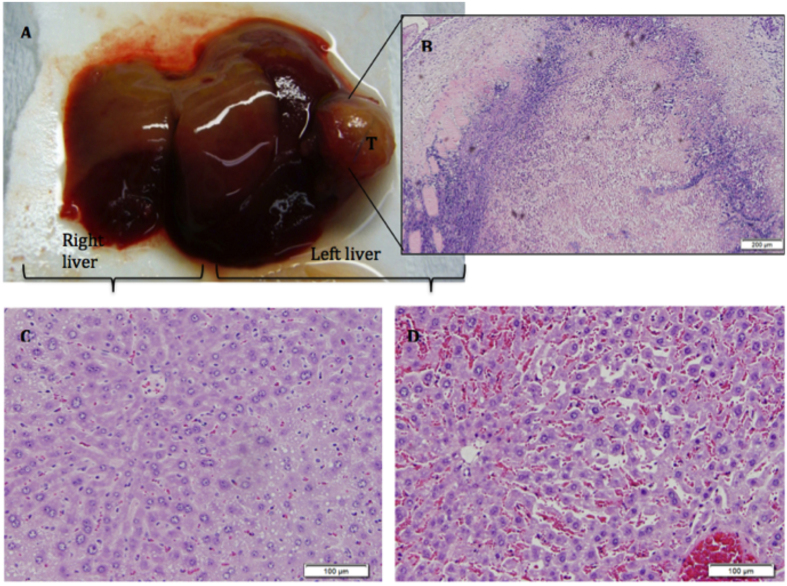
(**A**) Representative liver showing the macroscopic effect of electroporation in the treated left liver. (**B**) Extensive tumor necrosis after IRE is shown in microscope detail. Histological analysis of both liver lobes by HE histological staining revealed differences between the electroporated liver (**D**) and control (**C**).

The electroporated area of the liver showed infiltration of histocytes and lymphocytes, extensive areas of vascular congestion and interstitial oedema around hepatocytes, but no evidence of damage in the architecture of the hepatic sinusoids. Hepatic cells were surrounded by an eosinophil inflammatory infiltrate. The presence of fibrin deposits between the vascular layers and the observation of loose perivascular oedema on vessels confirmed that there was enhanced vascular permeability. Post-IRE tissue histological examination treatment of tumoral mice revealed extensive tumor necrosis (Fig. 2). In some of the tumors there were no signs of remnant tumoral cells while in other cases residual viable tumoral cells were still observed.

## Discussion

Our results revealed early mortality (<24 hours) in 47.2% of the total animals when any IRE protocol was applied involving a large liver volume and strong correlation between the electric field strength and this early mortality. However we were able to treat the diselectrolytemia and reduce the early mortality that occurred in 88% of the animals subjected to a 2000 V/cm field when no anti-hyperkalemia therapy was applied. This phenomenon has been observed in two completely different strains of mice with and without tumors.

Our hypothesis is that because of the increase in cell membrane permeability caused by electroporation certain potentially dangerous intracellular species freely diffuse from the intracellular medium to the extracellular medium. This phenomenon would be comparable to that of tumor lysis syndrome that occurs at the beginning of treatment with chemotherapeutics, in which tumor cells release intracellular products to bloodstream that overwhelm the excretory system^29^,^30^. In particular, we hypothesize that, in our study, released potassium ions were the major mortality cause as most deaths occurred within a few minutes after delivery of the pulsed electric fields. Potassium is one of the ions found in greater intracellular concentration^31^ (98% of the body’s potassium is in the intracellular fluid in a concentration of about 140 mmol/L). Cellular potassium levels are regulated by Na-K-ATPase and a passive leak mechanism driven by the electrochemical gradient favours potassium leaving the cell^32^.

It has been reported that potassium values over 6.5 mmol/L can cause abnormalities in the electrocardiogram (ECG) and lead to potentially fatal ventricular arrhythmias. As is evident in the results mean potassium levels in the lower survival group was 9.49 ± 4.11 mmol/L, which exceeds the upper limit of normality (serum potassium values in mice: 5.1–10.4 mmol/L)^28^.

We presume that the larger the ablation target volume, the greater the disturbances expected in the homeostasis, and hence the higher the risk of early mortality. Ball *et al*. demonstrated slight metabolic acidosis and hyperkalemia in 4 subjects from a group of 28 patients (14.3%) in which IRE was applied for ablation^19^. It was suggested that diselectrolitemia occurred in individuals who underwent ablation of very large tumors. However, this seems to be the only human study in which these alterations have been reported.

In a recent prospective multicenter study on 150 patients undergoing IRE − to our knowledge the largest human series published − high morbidity was found but no acid-base balance disorders were described^33^. In that study bipolar or monopolar probes were used to apply pulses, achieving a maximum ablation volume of 3.8 cm in diameter, which is probably not enough to produce the expected effects. Other studies in which IRE was used for hepatocellular carcinoma (CHC) ablation reported a maximum ablation diameter of 3.5 cm^34^. It has been described that the success rate of IRE rapidly decreases with the tumor size 12. In a recent systematic review, Scheffer *et al*. showed that, while from 67 to 100% primary efficacy was obtained in small lesions, efficacy dropped dramatically to 45% in large tumors^35^. It has been suggested that the cause is incomplete ablation of large tumors because of limited coverage by existing electrodes and generators^33^. Therefore, technological advances in ablating larger volumes with larger fields can be anticipated and dangerous metabolic acidosis and hyperkalemia could be manifested in the future.

Additional studies are required to consider this IRE approach. With improved surgical techniques and chemotherapy, surgically unresectable tumors are limited to large, multifocal tumors or to those located in difficult situations. Although IRE has been shown to avoid injury to the bile ducts and vessels^27^, little is known about its results when applied to large areas. In this context irreversible electroporation becomes both promising and a challenge.

To our knowledge this is the first study in which it is revealed that life threatening electrolytic disturbances occur after IRE ablation of a large portion (40%) of the liver. Although in current clinical practice such ablation volume is improbable, our results should serve as a warning to clinicians performing multiple or large ablations. Large liver ablations by IRE are being assayed in animal models and, presumably, will be tried in humans once the generators are able to apply the required electric fields and currents. It must be noted that the safety threshold remains unknown.

In conclusion, we consider that ionic imbalances produced by IRE are not an absolute contraindication for its use. Not certainly now, because the ablated volumes are too small, but also not in the future because, as our study shows, it will be possible to manage those imbalances. However, intensive patient monitoring, and in particular of his or her ionogram, will of course be necessary to treat possible imbalances caused by IRE, for which therapeutic approaches can be envisioned.

## Additional Information

**How to cite this article**: Sánchez-Velázquez, P. *et al*. Irreversible electroporation of the liver: is there a safe limit to the ablation volume? *Sci. Rep*. **6**, 23781; doi: 10.1038/srep23781 (2016).

## Figures and Tables

**Table 1:** Classification of the different groups of animals.

Mice strain	IRE Protocol (V/cm)	Anti-hyperkalemia therapy	N = 87	Survivors at 24 h	
				Amount	Survival days mean (range)
Tumoral athymic-Nude (ID)	SHAM	No	15	15 (100%)	**74.5 (49**–**122)**
	1000 V/cm	No	11	8 (72.7%)	**63.1 (2**–**121)**
	2000 V/cm	No	9	0 (0%)	—
		Yes	20	16 (80%)	**112.6 (28**–**176)**
C57-Bl6 (IC)	1000 V/cm	No	2	2 (100%)	*
	2000 V/cm	No	8	2 (25%)	*
		Yes	22	10 (45.5%)	*

*Animals of the non-tumoral group were euthanized after 7 days.

**Table 2:** Univariable and Multivariable analyses in mice of the group of 24 h-survival.

	Univariate			Multivariate		
	OR	95% CI	P	OR	95% CI	P
Postoperative hyperkalemia	1.4	1.1–1.7	0.01	1.4	1.0–1.9	0.028
IRE protocol 2000V/cm	4.7	1.8–11.8	0.001	17.8	2.5–125.6	<0.004
Medical treatment against hyperkalemia	0.1	0.02–0.32	<0.001	0.3	0.1–0.8	<0.023
